# Receptor-Binding-Domain-Specific B Cell Responses Induced by mRNA Immunization against SARS-CoV-2

**DOI:** 10.3390/vaccines11071148

**Published:** 2023-06-25

**Authors:** Maria Geropeppa, Ioanna Papadatou, Panagiotis Sarantis, Marianna Tzanoudaki, Ioannis Ntanasis-Stathopoulos, Tina Bagratuni, Evangelos Terpos, Vana Spoulou

**Affiliations:** 1 Immunobiology and Vaccinology Research Laboratory, School of Medicine, National and Kapodistrian University of Athens, 11527 Athens, Greece; 2 First Department of Pediatrics, School of Medicine, “Aghia Sophia” Children’s Hospital, National and Kapodistrian University of Athens, 11527 Athens, Greece; 3 University Research Institute for the Study of Genetic & Malignant Disorders in Childhood, “Aghia Sophia” Children’s Hospital, National and Kapodistrian University of Athens, 11527 Athens, Greece; 4 Molecular Oncology Unit, Department of Biological Chemistry, School of Medicine, National and Kapodistrian University of Athens, 11527 Athens, Greece; 5 Department of Immunology and Histocompatibility, Specialized Center and Referral Center for Primary Immunodeficiencies, Pediatric Immunology, “Aghia Sophia” Children’s Hospital, 11527 Athens, Greece; 6 Department of Clinical Therapeutics, School of Medicine, National and Kapodistrian University of Athens, 11528 Athens, Greece

**Keywords:** SARS-CoV-2, mRNA vaccines, immunological memory, antigen-specific memory B cells, neutralizing antibodies

## Abstract

mRNA vaccines have been instrumental in controlling the SARS-CoV-2 pandemic, but the short-lived protection mediated by Receptor Binding Domain (RBD)-specific antibodies necessitates frequent revaccinations to enhance vaccine-induced immunity. The development of RBD-specific B cell memory is critical for improving the qualitative and quantitative characteristics of the immune response. However, the effect of additional doses of mRNA vaccines on the composition of the RBD-specific B cell memory pool remains unclear. In this study, we found that dual BNT162b2 vaccination significantly increased both total RBD-specific and memory RBD-specific B cells and neutralizing antibodies. Following the second BNT162b2 dose, we showed a trend for the enrichment of CD27+IgM− memory RBD-specific B cells, which are known to correlate with a strong humoral response upon re-challenge. Repeated Measures Correlation (rmcorr) analysis revealed a significant correlation between antibody titers and both total and memory RBD-specific B cells, demonstrating that B cell and antibody responses are generated in a coordinated manner following BNT162b2 mRNA immunization. Our findings indicate that additional doses of the BNT162b2 mRNA vaccine enhance the qualitative and quantitative enrichment of the memory B cell pool against the vaccine antigens and collectively demonstrate the induction of a coordinated immune response to mRNA vaccination.

## 1. Introduction

The emergence of severe acute respiratory syndrome coronavirus 2 (SARS-CoV-2) has led to the development of messenger RNA (mRNA) vaccines, which have played a crucial role in controlling the coronavirus disease 2019 (COVID-19) pandemic by demonstrating an efficacy up to 95% in preventing severe COVID-19 [1]. Despite their success, questions remain regarding the immunological mechanisms responsible for inducing protective immunity, including the magnitude and longevity of the response. Reports of a rapid decline in antibody titers following immunization have prompted the need for frequent revaccinations to enhance the quantitative characteristics of humoral responses [2,3,4].

Antigen-specific B cells play a critical role in immunity following immunization, serving a dual function by producing antigen-specific antibodies through short-lived antibody-secreting cells while also establishing antigen-specific immunological memory. B cell immunological memory consists of two major cellular components: long-lived, high-affinity antibody-secreting cells that maintain a continuous immunoglobulin secretion, and memory B cells that are responsible for rapid recall responses [5]. The quality of the recall response is determined by the memory B cell repertoire, which undergoes affinity maturation and a class switch inside germinal center reactions [6]. Different subsets of memory B cells are considered to serve different functions in the recall response; IgM− memory B cells differentiate into plasma cells for the production of additional antibodies, while IgM+ memory B cells replenish the memory B cell pool by re-entering the germinal center reactions for the generation of new IgM+ and IgM− memory B cells [7,8]. Despite the significant role of memory B cells in generating protective immunity, it is still unclear how additional doses of mRNA vaccines affect the composition of the receptor binding domain (RBD) of the SARS-CoV-2-specific (RBD-specific) memory B cell pool, which represents the qualitative characteristics of the immune response.

We have previously shown that primary immunization with the Pfizer-BioNTech mRNA vaccine (BNT162b2) induces a coordinated response of the innate and adaptive immunity, with upregulation of the Type I interferon pathway and generation of Spike-protein-specific CD4 T cells that correlate with antibody responses to subsequent doses [9]. This suggests that additional BNT162b2 doses may improve the breadth of the T cell response, which could sustain the enrichment of the antigen-specific memory B cell pool and contribute to the qualitative characteristics of the recall response.

In our study, we utilized immunophenotyping of B cell subpopulations and measurement of antibody titers induced by the BNT162b2 mRNA vaccine, in order to investigate the effect of additional doses on the qualitative and quantitative characteristics of the B cell immune response, respectively.

## 2. Materials and Methods

### 2.1. Immunizations and Bleeding Points

Twenty-three SARS-CoV-2-naïve adults aged 30 to 85 years were recruited for the study between January and May 2021, after informed consent. Prior to enrollment, previous COVID-19 infection was excluded using the immunoassay Elecsys^®^ Anti-SARS-CoV-2 (Roche Diagnostics, Rotkreuz, Switzerland) for the semiquantitative detection of total antibodies (IgA, IgM, and IgG) against the SARS-CoV-2 nucleocapsid protein, following the manufacturer’s instructions. The demographic features of the participants are described in Table 1. Subjects with major comorbidities affecting the immune system (malignancies, immunosuppression, heart failure, chronic obstructive pulmonary disease, chronic kidney disease, liver failure, genetic syndromes) were excluded from the study. The participants were immunized with two doses of the BNT162b2 vaccine (Pfizer/BioNTech, Mainz, Germany), with a three-week interval. Peripheral blood was obtained for peripheral blood mononuclear cells (PBMCs) and sera isolation immediately before (Day 22, D22) and four weeks after the second dose of the vaccine (Day 50, D50).

### 2.2. Sera and Peripheral Blood Mononuclear Cells (PBMCs) Isolation and Storage

Sera were isolated and stored at −20 °C. PBMCs were isolated via density gradient centrifugation using density gradient medium (Lymphosep, Lymphocyte Separation Media, Biosera, Cholet, France). Isolated PBMCs were cryopreserved in 10% DMSO and 90% FBS and stored at −80 °C until batch analysis.

### 2.3. Antibody Analysis

Neutralizing antibodies (NAbs) against SARS-CoV-2 were measured with the cPas SARS-CoV-2 NAbs Detection Kit (GenScript, Piscataway, NJ, USA). This assay allows the indirect detection of total SARS-CoV-2 NAbs in the samples, by measuring the antibody-mediated inhibition of SARS-CoV-2 RBD region binding to its known human host receptor angiotensin converting enzyme 2 (ACE2). The percent signal inhibition for the detection of neutralizing antibodies was calculated based on the following formula: % signal inhibition = [1 − (optical density (OD) value of sample/OD value of negative control)] × 100, according to the manufacturer’s instructions.

### 2.4. Detection of RBD-Specific B Cells

Briefly, thawed PBMCs were stained with the following monoclonal antibodies: anti-CD19-APC-Vio-770, anti-CD27-VioBright, anti-IgM-APC, 7-AAD staining solution, and recombinant RBD-tetramer-PE and RBD-tetramer-PE-Vio-770 (SARS-CoV-2 RBD B Cell Analysis Kit, Miltenyi Biotec, Bergisch-Gladbach, Germany), according to the manufacturer’s instructions. For each sample, at least 10,000 B cell events were acquired (range 10,000–230,000, median 35,000) and a streptavidin-PE+streptavidin-PE-Vio-770+ negative control was used. An inclusion threshold of >30 RBD+ events in samples stained with RBD-tetramers was defined for the immunophenotyping of memory RBD-specific B cells. Flow cytometry data were acquired on a 3-laser 10-channel Navios Flow Cytometer (Navios, Beckman Coulter, Miami, FL, USA) and analyzed using Kaluza 2.1.1 software (Beckman Coulter). The gating strategy is detailed in Supplementary Figure S1.

### 2.5. Statistical Analysis

Continuous variables are described as median values. The D’Agostino–Pearson omnibus normality test was performed to test whether the values show a normal distribution. Paired samples were compared with the non-parametric Wilcoxon signed-rank test. All continuous variables were tested for correlations using the Spearman’s correlation coefficient. Correlations between variables with repeated measurements were assessed using the Repeated Measures Correlation in R [10]. All comparative analyses were performed with GraphPad Prism software (version 6.0) and R version 4.0.5 (31 March 2021). Statistical significance was set at *p*-value < 0.05 in all tests.

## 3. Results

### 3.1. Total and Memory RBD-Specific B Cell Immune Responses to Dual BNT162b2 Immunization

**Figure 1:**
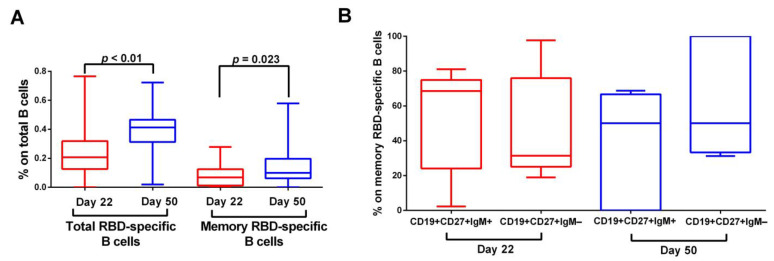
B cell response to dual mRNA immunization. (**A**) Total RBD-specific B cells and memory RBD-specific B cells and (**B**) Composition of memory RBD-specific B cell pool before (Day 22) and after (Day 50) the second BNT162b2 vaccine dose in SARS-CoV-2-naïve individuals. Comparisons were performed using the Wilcoxon matched-pairs signed rank test. Data are represented as median values (min to max).

During the study period, 23 SARS-CoV-2 naïve individuals were recruited for the purposes of our study. We first enumerated the total and memory B cells induced by dual BNT162b2 immunization. RBD-specific total B cells were generated following the first BNT162b2 dose and were significantly enriched on D50 (0.2075% vs. 0.4132% of total B cells, *p = 0.0027*) (Figure 1A). Memory RBD-specific B cells were also detected already after the first dose and were significantly increased after the completion of the two-dose schedule (0.06896% vs. 0.09965% of total B cells, respectively, *p = 0.0233*) (Figure 1A).

We further characterized the different subsets of the generated antigen-specific memory B cells, by analyzing their immune phenotype via Flow Cytometry. On D22, CD19+CD27+IgM+ memory B cells were the predominant cell phenotype over CD19+CD27+IgM− memory B cells (68.57% vs. 31.43% of memory RBD-specific B cells, *p = 0.8*) (Figure 1B). However, on D50, the frequency of CD19+CD27+IgM− memory B cells increased (CD19+CD27+IgM+ memory B cells: 50%, CD19+CD27+IgM− memory B cells: 50%, *p = 0.4*) (Figure 1B).

### 3.2. Serological Response to Dual BNT162b2 Immunization and Correlation between Antibody Titers and B Cell Response

**Figure 2:**
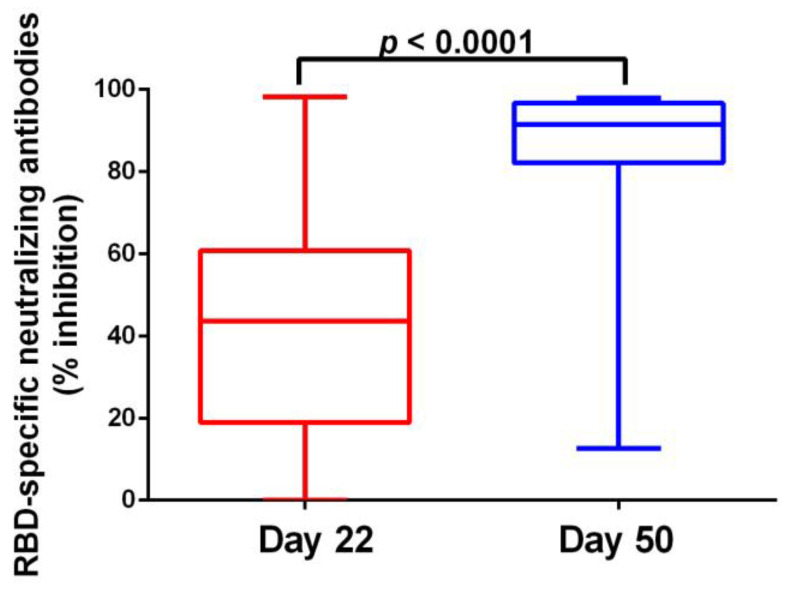
Serological response to dual mRNA immunization. Inhibition (%) of SARS-CoV-2 binding to the human host receptor angiotensin-converting enzyme-2 before (Day 22) and after (Day 50) the second BNT162b2 vaccine dose in SARS-CoV-2 naïve individuals. Comparisons were performed using Wilcoxon matched-pairs signed rank test. Data are represented as median values (min to max).

We next investigated the serological response of the participants on Days 22 and 50. RBD-specific neutralizing antibodies were detected already after the first vaccine dose on D22 and increased significantly four weeks after the second vaccine dose on D50 (43.63% vs. 91.56%, *p <* 0.0001) (Figure 2).

We then stratified the participants into high and low responders to immunization based on antibody titers achieved on Day 22. For that purpose, vaccinated individuals with <30% of RBD-specific neutralizing antibodies were considered low responders and individuals with >30% of RBD-specific neutralizing antibodies were considered high responders. After stratification for humoral titers, 13 participants were stratified into high responders and 10 participants were stratified into low responders. Total and memory RBD-specific B cells among high and low responders did not differ significantly on Day 22 (median values 0.208% vs. 0.184% of total B cells, *p* = 0.8; 0.05% vs. 0.07% of total B cells, *p* = 0.6, respectively) and on Day 50 (median values 0.423% vs. 0.377% of total B cells, *p* = 0.8; 0.11% vs. 0.08% of total B cells, *p* = 0.5, respectively).

**Figure 3:**
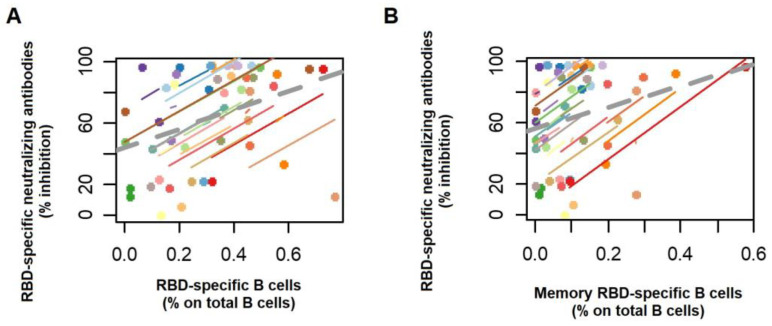
Serological and cellular components of B cell immunity are induced in a coordinated manner. RBD-specific neutralizing antibodies (NAbs) were correlated with (**A**) RBD-specific B cells and (**B**) memory RBD-specific B cells. The correlation was estimated using the Repeated Measures Correlation in R (rmcorr). Each participant’s data and corresponding line are displayed in a different color.

Finally, we investigated the correlation between humoral and B cell immune responses to BNT162b2. The Repeated Measures Correlation (rmcorr) demonstrated a significant correlation between antibody titers and both total and memory RBD-specific B cells (r = 0.511, *p* = 0.011 and r = 0.470, *p* = 0.02, respectively) (Figure 3).

## 4. Discussion

Our study demonstrates quantitative and qualitative enrichment of the antigen-specific memory B cell pool following secondary immunization with the BNT162b2 mRNA vaccine, with an increase in the total antigen-specific memory B cells and, within them, an enhancement in the switched (CD19+CD27+IgM−) memory B cell compartment where affinity maturation and a class switch occur.

Our findings are consistent with recent studies documenting the generation and enrichment of memory B cells after dual mRNA immunization [11,12], although subsets of B cells are presented in our study as percentages of total B cells and not as absolute lymphocyte numbers. As memory RBD-specific B cells are long-lived and persist longer than neutralizing antibodies [13], such findings could contribute to the longevity of protection against severe disease, which has been observed despite the rapidly declining neutralizing antibodies [13,14]. In our cohort of adults without immunocompromising conditions, the low immunogenicity seen in some subjects following the first dose of BNT162b2 (i.e., low responders) was overcome by an additional second dose.

Further phenotypic analysis of memory RBD-specific B cells demonstrated that while CD19+CD27+IgM+ memory B cells were the predominant cell phenotype after a single dose of the vaccine, the second dose of BNT162b2 induced an increase in CD19+CD27+IgM− memory B cells, up to 50% of total memory B cells. These findings indicate that the second dose leads to class-switch reactions known to be followed by an enhancement of somatic hypermutation and affinity maturation. Since CD19+CD27+IgM− memory B cells can rapidly generate an early burst of high-affinity antibodies upon secondary challenge, they may contribute, alongside the long-lasting T-cell immunity, to the observed reduction in disease severity in individuals who get infected despite previous vaccination [15]. The increase in CD19+CD27+IgM− memory B cells did not reach statistical significance, probably due to the small study size.

Regarding the serological response, similarly to previous reports, RBD-specific neutralizing antibodies were significantly increased after dual mRNA immunization [12,16,17]. Furthermore, we demonstrated a coordinated way of cellular and humoral B cell responses when the common intra-individual association for paired measures data was assessed. Collectively with our previous work [9], we now demonstrate that mRNA vaccines induce all arms of cellular and humoral adaptive immune including RBD-specific T and B cells and neutralizing antibodies in a coordinated way.

Despite the implementation of additional universal vaccinations as a countermeasure of the waning immunity against SARS-CoV-2, this policy may not be sustainable on a longitudinal basis. Considering the observed inter-individual differences in the duration of protection, it is crucial to identify low responders to vaccination using a convenient biomarker in order to optimize vaccination policies, as these vaccinated individuals could benefit most from additional vaccine doses. Since establishing antigen-specific B cells as a correlate of protection of SARS-CoV-2 vaccines may not be feasible in a real-world setting, due to the extensive cost and laborious nature of the Multicolor Flow Cytometry methodology, we propose that antibody titers before and after a vaccine dose could serve as an indicator for the detection of individuals who may not respond to immunization and may require additional vaccinations.

## 5. Conclusions

Collectively, our study highlights the effect of additional vaccine doses on the quantitative and qualitative improvement of the immune response to SARS-CoV-2 and provides further evidence on the underlying mechanisms of the immune response to mRNA vaccines by demonstrating the coordinated induction of cellular and humoral RBD-specific B cell responses.

## Figures and Tables

**Table 1:** The demographic characteristics of the enrolled participants.

*N*	23
*Age* Median (in years) Range (in years)	53 30–85
*Gender* Female (%) Male (%)	65.2 34.8
*Body Mass Index (BMI)* Mean (SD)	24.7 (4.1)

## Data Availability

Further information about data supporting the reported results will be provided by the corresponding author upon reasonable request.

## Supplementary Materials

The following supporting information can be downloaded at: https://www.mdpi.com/article/10.3390/vaccines11071148/s1, Figure S1: Gating strategy of RBD-specific B cells.
