# Supplementary material for: Receptor-Binding-Domain-Specific B Cell Responses Induced by mRNA Immunization against SARS-CoV-2
Source: Vaccines (Basel). 2023 Jun 25;11(7):1148. doi: 10.3390/vaccines11071148 (PMC10383073; doi:10.3390/vaccines11071148)
Supplement: Supplementary file 1 [file vaccines-11-01148-s001.zip › vaccines-2433104-Supplementary Figure S1.pdf]

SUPPLEMENTARY MATERIALS

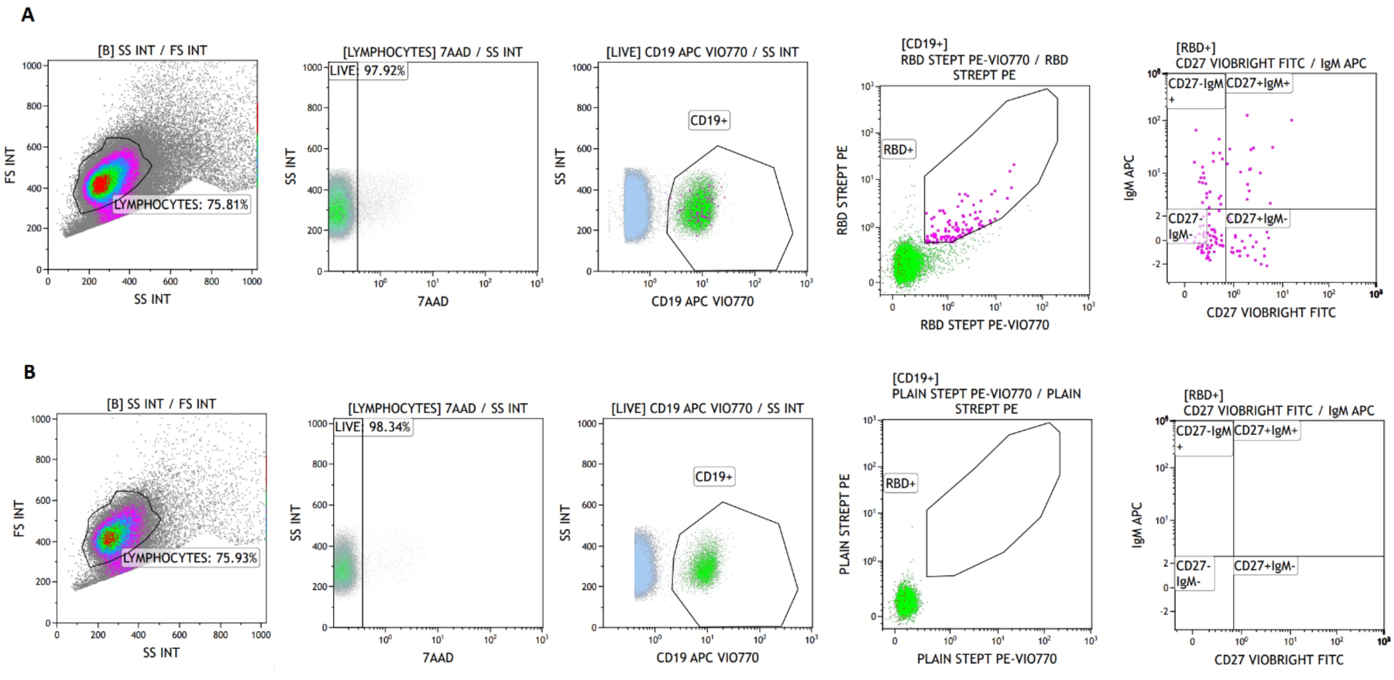

Supplementary Figure S1. Gating strategy of RBD-specific B cells

Flow cytometric characterization of SARS-CoV-2 RBD-specific B cells in peripheral blood mononuclear cells (PBMCs) isolated from SARS-CoV-2 naïve healthy adults immediately before and 28 days after the second dose of the mRNA BNT162b2 vaccine. RBD-stained (**A**) and negative control samples (**B**) are presented from the PBMCs of one of the study participants.
